# The Neurodevelopmental Pathogenesis of Tuberous Sclerosis Complex (TSC)

**DOI:** 10.3389/fnana.2020.00039

**Published:** 2020-07-14

**Authors:** David M. Feliciano

**Affiliations:** Department of Biological Sciences, Clemson University, Clemson, SC, United States

**Keywords:** TSC1, TSC2, mTOR, Tuber, SEGA, subependymal giant cell astrocytoma

## Abstract

Tuberous sclerosis complex (TSC) is a model disorder for understanding brain development because the genes that cause TSC are known, many downstream molecular pathways have been identified, and the resulting perturbations of cellular events are established. TSC, therefore, provides an intellectual framework to understand the molecular and biochemical pathways that orchestrate normal brain development. The TSC1 and TSC2 genes encode Hamartin and Tuberin which form a GTPase activating protein (GAP) complex. Inactivating mutations in TSC genes (TSC1/TSC2) cause sustained Ras homologue enriched in brain (RHEB) activation of the mammalian isoform of the target of rapamycin complex 1 (mTORC1). TOR is a protein kinase that regulates cell size in many organisms throughout nature. mTORC1 inhibits catabolic processes including autophagy and activates anabolic processes including mRNA translation. mTORC1 regulation is achieved through two main upstream mechanisms. The first mechanism is regulation by growth factor signaling. The second mechanism is regulation by amino acids. Gene mutations that cause too much or too little mTORC1 activity lead to a spectrum of neuroanatomical changes ranging from altered brain size (micro and macrocephaly) to cortical malformations to Type I neoplasias. Because somatic mutations often underlie these changes, the timing, and location of mutation results in focal brain malformations. These mutations, therefore, provide gain-of-function and loss-of-function changes that are a powerful tool to assess the events that have gone awry during development and to determine their functional physiological consequences. Knowledge about the TSC-mTORC1 pathway has allowed scientists to predict which upstream and downstream mutations should cause commensurate neuroanatomical changes. Indeed, many of these predictions have now been clinically validated. A description of clinical imaging and histochemical findings is provided in relation to laboratory models of TSC that will allow the reader to appreciate how human pathology can provide an understanding of the fundamental mechanisms of development.

## Introduction

The first clinical description of tuberous sclerosis complex (TSC) was of L. Marie, an infant that had seizures and intellectual delay, and later died at the age of 15. The physician Désiré-Magloire Bourneville described that L. Marie had a confluence of vesicular-papular eruptions and polyps on her skin (Brigo et al., 2018). She had hard, dense protuberances on the cerebral cortex, which he called *sclérose tubéreuse des circonvolutions cérébrales* (Bourneville, 1880). And there were lesions on her kidneys and small bumps that protruded into the ventricles of her brain. Bourneville and Édouard Brissaud later examined a four-year-old boy that had similar cortical protuberances, seizures, and learning difficulties (Poirier and Ricou, 2010; Brigo et al., 2018). They found growths contiguous with the ventricular walls and tumors in the kidneys. Bourneville surmised that the cortical protuberances were responsible for seizures and that the anatomical lesions and clinical symptoms were manifestations related to a disease. Therefore, it is Bourneville to whom credit is given for first describing the pathognomic features of TSC.

These early clinical observations provide several important theoretical contributions. First, that TSC involves numerous tissues. Patients have heart, kidney, and skin lesions, although currently, neurological issues remain most problematic. Notably, seizures occur in an overwhelming percentage of TSC patients and are likely caused by abnormalities within the cerebral cortex. Second, not all parts of every tissue are equally affected. Rather, discrete subsets of cells within a tissue are altered. As we will discuss later, these early observations provide evidence for somatic genetic mosaicism. Third, that although issues continue to arise throughout life for TSC patients, even at the earliest points, there are congenital anomalies and pathophysiological conditions consistent with the idea that TSC is a developmental disorder. It therefore should not be surprising to learn that as medical, genetic, and imaging technology have progressed, that TSC can be diagnosed *in utero*.

## Hamartin and Tuberin

The *TSC1* and *TSC2* genes encode for the proteins hamartin and tuberin that form a GTPase activating protein (GAP) complex and inhibit RAS homologue enriched in brain (RHEB; Garami et al., 2003; Inoki et al., 2003; Tee et al., 2003; Zhang et al., 2003). It is TSC2 that has the GAP domain and exhibits GTPase activating activity towards RHEB (Tee et al., 2003). TSC1 is required to stabilize TSC2 but does not have an intrinsic GAP activity. A third protein, TBC1D7, is bound to and required for the complex to function (Dibble et al., 2012). GTP bound RHEB activates protein kinase mammalian target of rapamycin mTOR complex 1 (mTORC1; Garami et al., 2003; Inoki et al., 2003; Tee et al., 2003; Zhang et al., 2003). Therefore, mutations that inhibit hamartin/tuberin function increase mTORC1 activity.

mTOR partitions into two molecular complexes, mTORC1 and mTORC2 (Saxton and Sabatini, 2017). mTOR forms a homodimeric complex that has a circular catalytic loop (Yip et al., 2010; Aylett et al., 2016). The mTOR dimers are held together by mTORC specific proteins. In the case of mTORC1, this protein is Raptor and for mTORC2, this protein is Rictor (Hara et al., 2002; Kim et al., 2002; Dos et al., 2004). mTORC1 and mTORC2 regulate distinct molecular pathways (Saxton and Sabatini, 2017). mTORC1 is most notable for stimulating anabolic mRNA translation through phosphorylation of p70S6 kinase and the eukaryotic initiation factor 4E binding protein (Burnett et al., 1998; Gingras et al., 1999; Garami et al., 2003). This signaling cascade increases mRNA translation of 5’ terminal oligopyrimidine tract containing RNAs and increases ribosome biogenesis (Thoreen et al., 2012). mTORC1 stimulates other anabolic pathways (Saxton and Sabatini, 2017). In contrast, mTOR inhibits catabolic processes including autophagy (Ganley et al., 2009; Jung et al., 2009; Kim et al., 2011). In this way, mutations in *TSC1/2* increase mTORC1 and notably, cell growth.

## TSC Neurological Features

Approximately 50% of TSC patients are developmentally delayed or have an intellectual disability (Krueger et al., 2013b). Ninety percent of patients have TSC-Associated Neuropsychiatric Disorders (TANDs) which includes behavioral, psychiatric, neuropsychological, and social/emotional processing issues (Krueger et al., 2013b; de Vries et al., 2018). A significant proportion of patients (~50%) are diagnosed with an autism spectrum disorder. An estimated 84% of patients have seizures, although ascertainment bias likely means that a smaller percentage of TSC patients have seizures (Kingswood et al., 2014, 2017; Nabbout et al., 2019). Hyperexcitability as measured by electroencephalogram (EEG) frequently appears in the first years of life manifesting as small spasms characterized by coincident loss of truncal tone and a sudden increase in tonicity leading to a head-bobbing motion (Lux and Osborne, 2004; Kelley and Knupp, 2018; Nabbout et al., 2019). These infantile spasms are accompanied by hypsarrhythmia and developmental delay. Hyperexcitability also manifests as focal seizures in TSC patients (Nabbout et al., 2019). The age of seizure onset (detection) and the types of seizures can differ between TSC patients. For example, 38.6% of patients had infantile spasms with the mean age at diagnosis being 0.4 years, whereas 67.5% had focal seizures that were diagnosed at 2.7 years, and 79% of patients exhibit hyperexcitability by 2 years of age (Nabbout et al., 2019). The severity, frequency, and age of seizure onset are correlated with intellectual disability and ASD diagnosis (Numis et al., 2011; Nabbout et al., 2019). While the cause of TANDs is still being explored, cerebral cortical malformations called tubers are the leading culprit for seizures in TSC.

## Defining a Cortical Tuber

**Figure 1:**
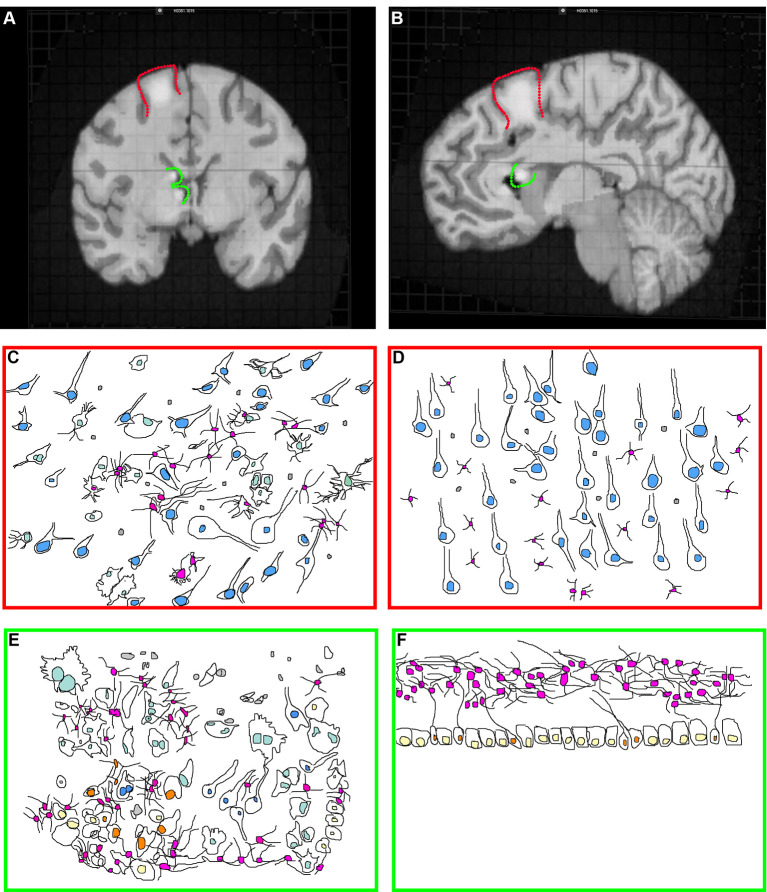
Tuberous sclerosis complex (TSC) histopathology. **(A)** Midsagittal section of an adult human brain MRI with a model cortical tuber (outlined in red) and subependymal giant cell astrocytoma (SEGA; outlined in green). Modified from the Allen Brain Atlas, The Brain Explorer 2 software. **(B)** Coronal section of an adult human brain with a model cortical tuber (outlined in red) and SEGA (outlined in green). Modified from the Allen Brain Atlas, The Brain Explorer 2 software. **(C,D)** Schematic of cortical tuber **(C)** and healthy **(D**; Mühlebner et al., 2016b) cortical tissue demonstrating neurons (blue nuclei), astrocytes (pink nuclei), microglia (gray), and giant cells (light green, tuber only). Note that cytomegaly, dysmorphic neurons, mislamination, gliosis, giant cells, and immune cell infiltration are features of “hot” epileptic cortical tubers. **(E,F)** Schematic of SEGA (Lopes et al., 1996) and healthy VZ-subventricular zone (SVZ) tissue (Sanai et al., 2011) from children demonstrating neurons (blue nuclei), astrocytes (pink nuclei), microglia (gray), ependyma (yellow), neural stem cells (NSCs; orange) and giant cells (light green, SEGA only).

Tubers are malformations detectable in ~80–90% of TSC patients and are classified as a major feature for TSC diagnosis (Krueger et al., 2013b; Kingswood et al., 2017). Tubers are most frequently found within the cerebral cortex but can occur elsewhere including the cerebellum (Doherty et al., 2005; Luat et al., 2007; Gallagher et al., 2010a; Kaczorowska et al., 2011; Mohamed et al., 2012; Pascual-Castroviejo et al., 2013; Vaughn et al., 2013; Boronat et al., 2017). TSC patients can have multiple anatomically distinct tubers of different size and location, and a single patient may have as many as 20 cortical tubers (Doherty et al., 2005; Luat et al., 2007; Gallagher et al., 2010a; Kaczorowska et al., 2011; Mohamed et al., 2012; Pascual-Castroviejo et al., 2013; Boronat et al., 2017). Cortical tubers can occur within the frontal, temporal, parietal, and occipital cortex (Qin et al., 2010b; Numis et al., 2011). Tubers appear by MRI (Figures 1A,B) as focal regions that have three categorically different changes in T1, T2, and weighted and fluid-attenuated inversion recovery (FLAIR) but are most obvious when hypointense in T1 and hyperintense on T2 weighted images (Gallagher et al., 2010a).

Focal epileptiform interictal discharges mapped by intracranial EEG frequently superimpose with cortical tubers (Mohamed et al., 2012). Tubers that have epileptiform activity are colloquially referred to as “hot tubers”. Hot tubers are targeted for surgical resection in cases of severe pharmacologically refractory epilepsy (Lachhwani et al., 2005). Tuber removal alleviates seizures in the majority of patients (Fallah et al., 2013, 2015). Intracranial EEG recordings demonstrate that seizure onset occurs within the core of the tuber and propagates toward the rim and eventually the area surrounding the tuber called the perituberal region (Kannan et al., 2016). Indeed, removal of the core has been demonstrated to be sufficient to alleviate seizures in some patients (Harvey et al., 2017). However, depth electrode recordings indicated that other tubers mapped by EEG may instead be electrophysiologically silent and that the perituberal region was responsible for epileptiform activity (Major et al., 2009). Indeed, surgical resection beyond tuber margins with the additional removal of perituberal region is beneficial for controlling seizures (Fallah et al., 2015). However, defining these regions by MRI is limited by imaging resolution. A recent study using microelectrodes, spiking activity, fast ripples, local field potentials, and intracranial EEG demonstrated that hyperexcitability is greatest within tubers (Despouy et al., 2019). To a lesser extent, changes are detected in the perituberal region, although not fast ripples (Despouy et al., 2019). Ultimately, this may reflect a limitation of current imaging technology, and the definition of what is a tuber can only be performed histopathologically. In support of this, histopathological studies on the TSC cortex demonstrates abnormalities that conventional imaging could not detect (Marcotte et al., 2012).

### Cortical Tuber Histopathology

Cortical tubers appear *in situ* as focal thickening of the cortex and can be cystic or calcified as a secondary response to epileptiform activity (Chu-Shore et al., 2009; Gallagher et al., 2010b; Zhang et al., 2018). Histological examination of cortical tubers supports their categorization into three groups based on cellular density and cell types present (Mühlebner et al., 2016b). Tubers are regions of cortical dysplasia for which the normally hexalaminar structure of the cortex is muddled (Figures 1C,D; Ferrer et al., 1984; Huttenlocher and Heydemann, 1984; Mühlebner et al., 2016b). Abnormal neurons are strewn throughout cortical layers, having either adapted the wrong identity or are ectopically/heterotopically positioned. Many times, neurons are enlarged (cytomegalic), shaped like balloon cells seen in focal cortical dysplasias, and can be dysmorphic. Abnormal neurons are intermixed with seemingly normal-looking neurons. Tubers can also have fewer neurons compared to surrounding tissue.

Tubers also contain giant-cells (Yamanouchi et al., 1997a,b; Mizuguchi and Takashima, 2001; Mizuguchi et al., 2002). Giant-cells are cytomegalic (enlarged) cells of unknown origin and function. TSC lesions including subependymal giant cell astrocytomas (SEGAs) also have giant cells (Hirose et al., 1995). Pathologists can consistently point to multiple types of giant-cells. For example, giant-cells can be neuron- or astrocyte-like (Hirose et al., 1995). Also, giant-cells of an intermediate/mixed morphology expressing markers of neural stem cells (NSCs) including nestin, vimentin, and SMI 311 or neuroblast markers such as doublecortin are identified (Crino et al., 1996; Mizuguchi et al., 2002; Mizuguchi, 2007). A subpopulation of tuber cells is multi-nucleated. These results point to the fact that cortical maturation is perturbed in tubers.

Pro-inflammatory molecules are also increased in epileptic tubers (Maldonado et al., 2003; Boer et al., 2010; Mühlebner et al., 2016b; Martin et al., 2017; Mills et al., 2017). Not surprisingly, there is immunological infiltration as demarcated by increased T-cells and activated microglia (Martin et al., 2017). The role of inflammation and immune invasion in the pathogenesis of TSC is unclear but it is certainly a component of hot tubers. Likewise, the role of astrocytes in TSC associated epileptogenesis is still unclear. There is reactive gliosis, increased glial fibrillary acidic protein (GFAP), and a reduction in glutamine synthetase in surgically resected tubers (Sosunov et al., 2008). A change in TSC astrocytes is also supported by mouse models that also demonstrate decreased glutamate transporter and Kir4.1 inward rectifying potassium channel expression (Wong et al., 2003; Jansen et al., 2005; Zeng et al., 2007, 2010; Xu et al., 2009; Wong and Crino, 2012). Many changes seen in TSC astrocytes can be evoked by seizure-inducing stimuli (Zhang and Wong, 2012). This is exemplified in mice with neurons lacking *Tsc1*, which induces secondary changes in astrocytes (Crowell et al., 2015). GFAP-CRE is active in NSCs thus some neurons in this model also have Tsc1 deleted (Zou et al., 2017). And at least in some cases, the deletion of *Tsc1* genes in mice does not activate astrocyte mTORC1 pathway activity (Feliciano et al., 2011; Carson et al., 2012). Therefore, additional work is needed to further establish the contribution of astrocytes to TSC epileptogenesis.

The cellular heterogeneity and mosaic patterning of abnormal cells surrounded by normal cells in tubers complicate both the analysis and conclusions based on surgically resected tubers. For example, cells reactive to abnormal developmental structuring or electrical hyperexcitability may not have primary roles in the pathogenesis of TSC but are included in analyses when a tuber is removed. Hot vs. cold tuber studies have revealed important differences, but cold tuber vs. control tissue experiments are less frequently performed (Bagla et al., 2018). Studying hot vs. non-epileptic cold tubers could determine whether changes in astrocyte, microglia, T-cells, or blood vessels are secondary responses to focal seizures or a driver of epileptogenesis. Gene ontology and cellular deconvolution may also help reveal changes to specific cell types within tubers and differentiate among these possibilities. However, single-cell RNA sequencing of resected TSC lesions is a needed future direction, in part to separate normal and abnormal cells in the mosaic tuber.

Increased α-[(11)C]-methyl-l-tryptophan (AMT) uptake by hot tubers located by MRI and validated hyperexcitability by EEG mapping demonstrates that tryptophan uptake and serotonin synthesis or kynurenine pathway activity may be increased during seizures (Chugani et al., 1998; Asano et al., 2000; Kagawa et al., 2005). Provocatively, these changes are also associated with the miRNA profile of cortical tubers (Bagla et al., 2018). Surprisingly, however, there have been few basic science studies looking at the link between tryptophan uptake and TSC. The major tryptophan transporter is comprised of Slc7a5 (LAT1) which is increased in TSC (Lim et al., 2011). And because LAT1 also transports amino acids that activate the protein kinase mTORC1, this transporter may be an important target for the development of future TSC treatments (Nicklin et al., 2009).

Immunohistochemical studies on tubers have revealed an array of changes that might affect neuron physiology. Neurons have altered AMPA receptor (AMPAR) and NMDA receptor (NMDAR) profiles (White et al., 2001; Talos et al., 2008). There are also significant reductions in GABA receptors in tubers(White et al., 2001; Talos et al., 2012). Moreover, NKCC1 chloride importer levels are increased whereas KCC2 chloride exporters are decreased (Talos et al., 2012). Therefore, the reversal potential of chloride is altered leading to GABA having a depolarizing effect on neurons. However, the sum effect of reducing GABA receptors and increasing NKCC1/KCC2 ratios is unclear. Promising mechanistic studies on model organisms and induced pluripotent stem cells are helping to elucidate which of these molecules are drivers of epileptogenesis (Auerbach et al., 2011; Bateup et al., 2013; Kelly et al., 2018).

## Cortical Tuberigenesis

The process of primary neurulation generates the primitive ventricular system by forming the neural tube (Schoenwolf and Smith, 1990). During this process, ectodermal cells of the trilaminar disc receive inhibitory cues that prevent their acquisition of an epidermal fate (Jessell and Sanes, 2000). This in turn allows for the generation of neuroepithelial cells (NECs). The pseudostratified layer of NECs invaginates to generate a fold that eventually fuses with itself. The closure of this fold forms the neural tube and creates a primitive ventricular system that is surrounded by NECs.

The region directly surrounding the ventricles is called the ventricular zone (VZ). The subventricular zone (SVZ) is located adjacent to and basally toward the VZ. Specialized mitotic NSCs derived from NECs called radial glia (RG) are found at dorsal and dorsal lateral regions of the telencephalic lateral ventricles (Rakic, 1988). RG has a soma seated near the ventricles that projects a basal fiber (Rakic, 1988). The basal fiber projects outward toward the pia or developing surface of the cortical plate (Rakic, 1988). RG produce neurons, astrocytes, oligodendrocytes, and ependyma in a temporally encoded manner. First, RG divides and generate neuroblasts that migrate radially along with the basal fiber toward the pial surface. The layers form in an inside-out fashion. This means that the deep layer (VI) neurons are generated first, followed by V, VI, III, and II. These excitatory neurons have different morphological, electrophysiological, and functional properties.

Human VZ RG produces an additional large population of outer (o) SVZ (basally produced) RG (Hansen et al., 2010). This unique and recently evolved adaptation expands substantially between 11.5 and 17 weeks of gestation, so much so that oSVZ RG is more numerous and divide more frequently at mid-gestation than VZ RG (Rash et al., 2019). VZ RG of rodents begin to generate astrocytes after neurogenesis, late in embryogenesis. In macaques, the oSVZ predominantly begins to generate glia by ~13 weeks (Rash et al., 2019). The marked increase and protracted period of gliogenesis caused by the oSVZ RG increase the total number of astrocytes in the cortex and is correlated with an evolutionary switch from a lissencephalic to gyrencephalic brain. The role of oSVZ RG in cortical development has raised the question of what pathways are required for oSVZ RG function. To address this question, single-cell RNA sequencing was performed and demonstrated that human oSVZ RG are relatively enriched in mTORC1 pathway genes and stain positive for phosphorylated S6 (Nowakowski et al., 2017). The importance of mTORC1 signaling oSVZ RG is also demonstrated by the fact that human primary cells and organoid cultures have elevated mTORC1 transcript levels and mTORC1 activity in VZ-oSVZ RG compared to macaques and chimpanzees (Pollen et al., 2019).

An additional group of NSCs is found around the medial and lateral ganglionic eminences surrounding the ventricles and give rise to GABAergic inhibitory neurons (Wamsley and Fishell, 2017). Inhibitory NSCs that express Nkx2.1 produce neuroblasts that migrate tangentially through the cortical plate or rostrally to the olfactory bulb (Butt et al., 2008).

What do tubers tell us about normal development? The first is that neurons are mislocalized in tubers. Mislocalization of mutated cells could be explained by aberrant migration or incorrect laminar fate choice. Cortical tuber dysplastic neurons and balloon cells found scattered throughout the cortical plate retain markers (Satb2, Cux2) of upper-layer neurons supporting the notion that it is the migration that is altered (Mühlebner et al., 2016a). Also, mouse models generated by conditional TSC deletion demonstrate that neurons destined to upper layers retain upper layer markers but are found in deeper layers of the cortex (Way et al., 2009; Moon et al., 2015). This is in agreement with migration assays in cells of the rostral migratory stream (RMS) that indicate TSC neuroblasts migrate slower (Feliciano et al., 2012). Cell-autonomous effects on migration are supported by the fact that neuroblast specific expression of constitutively active Rheb (an mTOR activator) is sufficient to induce cortical lamination defects (Lafourcade et al., 2013; Moon et al., 2015; Hsieh et al., 2016; Lin et al., 2016). One mechanism that may contribute to defective migration comes from studies of patients with activating mTOR mutations that cause cortical malformations (Park et al., 2018). *In utero* electroporation of mutant mTOR prevented cortical lamination, induced cytomegaly, and caused seizures in mice (Park et al., 2018). These changes were caused by inhibition of autophagy and defective ciliogenesis (Park et al., 2018). Importantly, autophagy and ciliogenesis were perturbed in patient samples including from TSC patients (Park et al., 2018). Mislocalized neurons do not need to be mutated, but rather can be effected through non-cell-autonomous mechanisms by TSC neurons (Moon et al., 2015). Significantly, cortical tubers can have fewer neurons (Mühlebner et al., 2016b). But to what extent the decrease is caused by faulty migration, reduced neurogenesis, or increased cell death is unclear. *Tsc2* knockout and long term *Tsc1* knockout mice stress neurons potentially leading to their loss (Di Nardo et al., 2009; Tsai et al., 2012; Reith et al., 2013). Another cause of neuron loss is that *TSC* mutations may prevent NSCs from creating neurons and instead increase gliogenesis as has been demonstrated in mouse models and patient derived induced pluripotent stem cells (Way et al., 2009; Magri et al., 2011; Blair et al., 2018).

## Modeling TSC Tubers

*Tsc1* and *Tsc2* homozygous mutant mice and rats die embryonically (Rennebeck et al., 1998; Onda et al., 1999; Kobayashi et al., 2001). Although NECs of *Tsc2* heterozygous mice are no different than controls, homozygous *Tsc2* NECs have robust transcriptional changes, up-regulate GFAP, and are defective in neural differentiation (Onda et al., 2002). The creation of mice having *Tsc1* or *Tsc2* genes flanked by lox P sites has circumvented the limitation of embryonic lethality and by allowing CRE mediated deletion of *TSC* genes from distinct cell populations (Kwiatkowski et al., 2002; Hernandez et al., 2007). *hGFAP, Emx1*, and *Nestin* promoter-driven CRE mediated *TSC1* gene deletion in RG and RG progeny causes mislamination, macrocephaly, cytomegaly, hypomyelination, and reactive gliosis with seizures (Way et al., 2009; Goto et al., 2011; Magri et al., 2011; Mietzsch et al., 2013). *Tsc2* deletion by *hGFAP-CRE* also generates macrocephalic mice having cytomegaly, hypomyelination, reactive gliosis, and seizures (Way et al., 2009; Mietzsch et al., 2013). Importantly, hGFAP-CRE *Tsc2* deletion has negligible effects on RG indicating that the phenotypes seen may have more to do with RG progeny (Way et al., 2009). In contrast, Emx1-Cre deletion of *Tsc1* increased Pax6 NSCs and BrdU, NSCs self-renew less efficiently and produce more GFAP positive cells at the expense of neurons (Magri et al., 2011). A common critique of these models was that no apparent cortical tubers were present owing to the ubiquitous removal of TSC genes in all progenitors of the forebrain. A complementary approach utilized *in utero* electroporation only affects a subset of cells in the developing brain resulting in a focal mosaic patterning, cytomegaly, and hyper-active mTORC1 similar to that seen in a patient in tubers (Feliciano et al., 2011). *Tsc1* postmitotic neural deletion starting at E12.5–13.5 caused similar effects in neurons that can be rescued with rapamycin and support the idea that TSC genes are critical for neuronal function (Meikle et al., 2007, 2008). The loss of *Tsc1* in late post-mitotic neurons using Camk2a-CRE mice also leads to seizures, albeit much later ~5–10 weeks (McMahon et al., 2012). The late loss of *Tsc1* inhibited autophagy which may prevent dendrite spine pruning (McMahon et al., 2012; Tang et al., 2014). Thus, the loss of TSC genes in NSCs or astrocytes, altered proliferation, and mislamination may not be required for epileptogenesis. In agreement, the electroporation of an inducible constitutive form of Rheb which is expressed at postnatal day six causes seizures at 2 months (Hsieh et al., 2016). Therefore, hyperexcitability can be caused by changes during neuroblast-neuron maturation. In agreement, Tsc1/2 expression is high within the cortical plate and maturing neurons (Li et al., 2018).

### The Effects of TSC1/2 Deletion on Neurons

How TSC neurons contribute to hyperexcitability remains an active area of interest. First, axon development is suppressed in cultured hippocampal neurons upon over-expression of hamartin and tuberin/hamartin reductions increase the number of axons (Choi et al., 2008). Another study however demonstrated that *in vivo* electroporation of constitutively active Rheb expedited axon growth (Gong et al., 2015). *Tsc1* deleted Purkinje cells of the cerebellum have spurious axon projections too, but also have increased dendritic spine density, and a reduced spiking rate (Tsai et al., 2012). Hypothalamic POMC neurons having *Tsc1* deleted have reduced firing rates too, a hyperpolarized resting membrane potential, and enhanced ATP-sensitive potassium current whereas *Tsc1* deleted AGRP neurons do not exhibit these changes (Yang et al., 2012). In contrast, dopamine D1 receptor-expressing striatonigral neurons are more excitable and exhibit higher firing rates but not D2 receptor-expressing striatopallidal neurons (Benthall et al., 2018). E12.5 GBX2-CRE *Tsc1* knockout generated neurons that exhibit improper axon projections, lower input resistance, higher capacitance, and changes to action potential dynamics that correspond to more rapid intraburst spiking (Normand et al., 2013). These results demonstrate cell-type specific effects on intrinsic excitability.

Incidentally, many studies to date have determined that network activity is likely altered by postsynaptic mechanisms. TSC neuron dendrite arbors are often significantly increased as are neuron somas (Meikle et al., 2008; Feliciano et al., 2011, 2012; Goto et al., 2011; Zhang et al., 2014; Blair et al., 2018; Kosillo et al., 2019). In contrast, although somas are enlarged in *Tsc1* deleted striatonigral neurons they have reduced arbors (Benthall et al., 2018). TSC neuron intrinsic pre-synaptic activity does not always change, and in the hippocampus, there is a larger size of soma and dendrite arbors, increased capacitance, and reduced electrical input resistance (Bateup et al., 2011, 2013). *Tsc1* knockout hippocampal neuron AMPAR and NMDAR-mediated current amplitude is elevated in *Tsc1* KO neurons and abolishes mGluR mediated long term depression (Bateup et al., 2011). Also, TSC neurons have fewer spontaneous excitatory postsynaptic currents and miniature excitatory postsynaptic currents (Bateup et al., 2013). This may be an effect of overall network activity being increased leading to compensatory transcriptional changes including reductions in AMPAR and loss of NMDA mediated long term potentiation. To overcome the limitation of network activity, sparse *Tsc1* knockout was performed and led to the conclusion that reduced inhibitory postsynaptic currents likely underlies the hyperexcitability, at least in hippocampal neurons. These studies demonstrate the complexity of distinguishing between primary mechanisms of hyper-excitability and secondary adaptations to that hyper-excitability, both of which are critical for understanding and treating TSC.

## Defining TSC Subependymal Nodules (SENs) and Subependymal Giant-Cell Astrocytomas (SEGAs)

TSC SENs are growths that emanate from the region surrounding the brain’s ventricles called the subependymal zone or SVZ. SENs are present in the majority (~85%) of TSC patients (Northrup et al., 1999; Hasbani and Crino, 2018). They are found near the caudate nucleus contiguous with the lateral ventricles but also near the Foramen of Monro leading to the third ventricle. TSC SENs arise embryonically but also during the neonatal period (Chan et al., 2018). SENs are classified as a major feature for the diagnosis of TSC (Krueger et al., 2013b).

Approximately 20% of TSC SENs transform into subependymal giant cell astrocytomas (SEGAs; Figures 1A,B; Northrup et al., 1999; Adriaensen et al., 2009; Kingswood et al., 2017; Chan et al., 2018). SEGAs are a type I benign neoplasia. They should not be confused with astrocytomas. SENs greater than 10 mm is considered SEGAs, but an increased growth rate as determined by a sequential MRI scan is the greatest criterion given for distinction of the two (Northrup et al., 1999). Histological profiles of TSC SEGAs and SENs overlap and there is currently no molecular marker that distinguishes between the two. TSC SEGAs can block the flow of cerebrospinal fluid (CSF) in the ventricular system which may lead to hydrocephalus, increased intracranial pressure, seizures, and can lead to death (Northrup et al., 1999; Adriaensen et al., 2009; Hasbani and Crino, 2018).

It is important to note that TSC2 mutations are associated with severe brain lesions and poor prognosis (Northrup et al., 1999; Hasbani and Crino, 2018). TSC2 mutations are overwhelmingly more frequently associated with SEGA formation. For example, a study demonstrated out of 207 patients with SEGAs, only 22 patients had TSC1 mutations whereas 185 had TSC mutations (Kingswood et al., 2017). It is important to remember that tuberin is the catalytic part of the TSC complex and tuberin and hamartin have biochemical, physiological, and clinically relevant differences.

### SEGA Histopathology

TSC SEGAs typically have few mature NeuN positive cells (Zordan et al., 2018). This is supported by the fact that MAP2 positive mature neuron labeling is sparse (Lopes et al., 1996). Patient SEGA analysis using antibodies such as beta III tubulin has been used to indicate that some samples have neuronal cells (Lopes et al., 1996). However, beta III tubulin also stains immature neurons (neuroblasts) that are present within these regions (Sanai et al., 2011). Another group found no neuronal neurofilament or synaptophysin staining in SEGAs but they do report neurons (You et al., 2005). That few synaptophysin positive neurons were identified is supported by a recent review that calls synaptophysin staining “patchy” (Cotter, 2019).

Giant cells are a hallmark of human TSC SEGAs (Figures 1E,F; Kwiatkowski and Manning, 2014). The presence of giant cells with an overlapping phenotype (both neuron and astrocyte) is documented. However even in SEGAs, cells of the overlapping phenotype are infrequent, and their importance/role is unknown. Staining is typically not performed simultaneously in clinical samples meaning that it is not clear that the antibodies stain the same precise cells. Nevertheless, it is not known whether there are overlapping glial/neural phenotypes or whether SEGAs contain both glia and neurons is not clear. Yet, both indicate that NSCs may contribute to SEGA pathogenesis. It is important to note that NSCs express many of the same marker proteins seen in glia (astrocytes). Many experts refer to the NSCs in the region as SVZ astrocytes (Wang and Bordey, 2008). One example of a marker protein having this pattern is the protein GFAP. Human NSCs around the ventricle and astrocytes in the lower cortical plate express GFAP under normal conditions. TSC SEGAs contain variable amounts of GFAP and have a glial fibrillary matrix (Lopes et al., 1996). One manuscript has reported that GFAP was not abundant in their samples (Nakamura and Becker, 1983). Another found GFAP staining, but that giant cells were not frequently GFAP positive (Debiec-Rychter et al., 1999). Therefore, it is not clear whether GFAP cells are astrocytes, NSCs, or a cell of completely different origins. Staining for GFAP, Nestin, GLAST, and SOX2 demonstrates that SEGAs contain NSC markers (Phi et al., 2008). It should be noted, however, that by the time most SEGAs are removed, years have gone by, patients frequently have severe seizures, and are often given a wide range of medicines to control seizures, neurological manifestations, and peripheral problems. Therefore, the analysis of surgically resected SEGAs is confounded by secondary changes.

## TSC SEGA-Genesis

Ventricular SENs/SEGAs in TSC can be identified by midgestation (Park et al., 1997; Mühler et al., 2007; Dragoumi et al., 2018). There are few examples of detailed analysis of TSC fetal tissue in the scientific literature (Park et al., 1997; Prabowo et al., 2013; Parker et al., 2014). A shared set of samples described in two manuscripts provides evidence of nodular lesions that can be detected as early as 22 gestational weeks (GW; Prabowo et al., 2013; Parker et al., 2014). Tissue from 23 GW twins confirmed the presence of subcortical and cortical plate lesions. Interestingly, this case also identified a region that was reminiscent of a SEGA. The tissue stained positive for Vimentin, GFAP, and Nestin, which in theory labeled NSCs. Given the location of these cells and the developmental time point, the identification of NSCs as the predominant cell type is predictable. Also, these tissues lacked staining for neuron markers (although there was one taken from an older case at 38 GW). Unfortunately, however, it is not clear whether this anomaly arose from NSCs or from astrocytes or even alternative cell types such as ependyma (a multi-ciliated cell that lines the ventricles throughout postnatal life). Examples of outer SVZ lesions were also found within 27 GW and 32 GW tissues. Moreover, abnormal enlarged balloon cells were identified within the cortical plate. These balloon cells were scattered in discrete regions. These results demonstrate that TSC SENs can begin during embryonic development.

The ventricular system is fluid-filled, containing CSF. CSF flows from the lateral ventricles (one in each hemisphere) through the Foramen of Monro into the medial-ventrally located third ventricle within the diencephalon found between the hypothalamic hemispheres (Lehtinen et al., 2013). CSF then flows through the cerebral aqueduct into the fourth posterior ventricle near the pons and medulla oblongata and eventually to the spinal cord and subarachnoid space (Lehtinen et al., 2013).

NSCs of the neonatal SVZ normally persist until 18 months of age (Sanai et al., 2011). SVZ NSCs generate glia that migrates into the lower cortical plate and neuroblasts that migrate rostrally along the RMS to the olfactory bulb and mature into neurons (Lim and Alvarez-Buylla, 2016). In humans, the RMS bifurcates and neuroblasts migrate through the medial migratory stream into the ventromedial prefrontal cortex (Sanai et al., 2011).

TSC SENs and SEGAs are also identified in neonates (Kotulska et al., 2014). The majority of SENs are detected by 2.5 years and nearly all before the age of 5 years (Kingswood et al., 2017). Since SENs are proposed to be asymptomatic, they are not targeted for surgical resection. Therefore, detailed immunohistochemical findings are unknown. One must note that although SENs are proposed to be asymptomatic, their prevalence in TSC is high as are many neurological manifestations of unknown etiology. Studies, for example on brain tumors, have revealed non-cell-autonomous mechanisms that lead to circuit changes (Buckingham et al., 2011; Yu et al., 2020). Therefore, in theory, SENs could play a role in the pathogenesis of certain neurological manifestations. It is also interesting to consider that SENs/SEGAs do not typically spontaneously arise after age 5 (Kingswood et al., 2017). This is in contrast with the typical relationship between advanced age and the development of cancers.

The evolution from SEN to SEGA in TSC is a gradual process typically confined to younger patients with the median age of SEGA identification of 8 years (Kingswood et al., 2017). In contrast, tumors typically occur more frequently later in life owing to an increased mutational burden which differs from TSC SEGAs. The formation of SEGAs therefore may not depend on the acquisition of secondary mutations of non-TSC genes, but rather stochastic mechanisms related to mTORC1. This might explain why the mitotic index of SEGAs is low and why SEGAs only occurs in a minority of TSC patients. Beyond a low mitotic index, another hint about the mechanism is that while much of the lesion is comprised of NSCs, neuron-like cells, and gemistocytic astrocyte-like cells. Therefore, the cell of origin may be multipotent.

### Modeling TSC SEGAs

Based on the timing of appearance, the anatomical location, and the cellular composition, it has been hypothesized that SVZ NSCs are the cell of origin of TSC SEN-SEGAs. To test this hypothesis, conditional *Tsc1* mice were crossed to tamoxifen-inducible nestin-CRE mice or subjected to single-cell neonatal SVZ NSC electroporation of CRE (Feliciano et al., 2012). Contemporaneous experiments used the same approach of conditional *Tsc1* mice crossed to tamoxifen-inducible *Nestin*-CRE or *Ascl1*-CRE mice (Zhou et al., 2011). The results of these experiments were that *Tsc1* deletion caused mTORC1 hyper-activation, neuronal heterotopias, and small ventricular lesions reminiscent of SENs. This is also relevant because similar lesions occur throughout the olfactory tract of TSC patients (de León et al., 1988; Ridler et al., 2004; Manara et al., 2018). However, well-defined SEGAs were not generated in these mouse models.

A recent manuscript demonstrated that double knockout of the tumor suppressor *Pten* and *Tsc1* in neonatal but not adult NSCs causes SVZ tumors in mice (Zordan et al., 2018). Yet *Pten* deletion and p53, NF1, or Ink4a alterations also cause SVZ tumors (Kwon et al., 2008; Zheng et al., 2008; Kim et al., 2012; Alcantara Llaguno et al., 2015). *Pten/Tsc1* deletion tumors may reflect the potent tumor suppressor functions of PTEN. Evidence has not yet linked the loss of a TSC gene and a second non-TSC gene in patient SEGAs (Henske et al., 1997; Chan et al., 2004; Bongaarts et al., 2017). Moreover, this same group did not find evidence that PTEN is mutated in SEGAs (Zordan et al., 2018). However, this raises the possibility that there may be genetic or environmental modifiers that promote SEGA formation.

*Tsc2* deletion causes more severe phenotypes than *Tsc1* in mice, but have only recently been performed in embryonic NSCs and GFAP positive postnatal astrocytes (Way et al., 2009; Zeng et al., 2011; Mietzsch et al., 2013; Moon et al., 2015). Recently, a group demonstrated that TSC patient tubers stain positive for excitatory NSC forebrain markers, that SEGAs stain positive for inhibitory NSC markers, and that inhibitory NSC *Tsc2* deletion generates lesions recapitulating SENs (Rushing et al., 2019).

## The Molecular Genetics of TSC

Blood lymphocyte DNA from families having multi-generational TSC inheritance was used to perform genetic linkage analysis. These experiments first linked TSC to chromosome 9 (Fryer et al., 1987). However, locus heterogeneity identified by subsequent studies determined a second locus on chromosome 16 (Sampson et al., 1989; Janssen et al., 1990; Haines et al., 1991; Povey et al., 1994). Mutations were mapped to 9q34.13 and 16p13.3 and called *TSC1* and *TSC2*, respectively (European Chromosome 16 Tuberous Sclerosis Consortium, 1993; van Slegtenhorst et al., 1997). Identification of mutations in *TSC1* or *TSC2* that prevent the production or function of their protein products (estimated by nonsense mutations, truncations, etc. or that are verified as non-functional) is sufficient for the diagnosis of TSC (Northrup et al., 1999; Hoogeveen-Westerveld et al., 2012, 2013). Approximately 75–85% of patients having clinical features sufficient for a TSC diagnosis have mutations in *TSC1* or *TSC2* that can be identified by routine genetic screening (Northrup et al., 1999; Dabora et al., 2001; Sancak et al., 2005; Au et al., 2007).

Familial TSC is often described as an autosomal dominant disorder following Mendelian inheritance patterns. However, there are limitations to this proposed model of pathogenesis. First, is that the discrete lesions form and hint at a more nuanced mechanism of pathogenesis. Second, mutation or deletion of TSC genes is embryonic lethal in rodent models (Rennebeck et al., 1998; Onda et al., 1999; Kobayashi et al., 2001; Murakami et al., 2004). Therefore, inherited dominant-negative mutations are presumed lethal. Third, TSC patients have phenotypic variability, even when comparing patients with the same affected gene. An interwoven tapestry of clinical observation, genetic screening, and laboratory research has addressed these limitations.

TSC patients have tumors including SEGAs, rhabdomyomas, renal cell carcinoma, angiomyolipomas, and lymphangioleiomyomatosis. In 1971, Knudson (1971) proposed the two-hit hypothesis to describe why some children with retinoblastoma have tumors in both eyes whereas others have tumors in one eye. Statistical modeling prompted him to hypothesize that a familial form of retinoblastoma arises when one copy of a gene is inherited (for example, from a father), and then a second mutation occurs in the other copy (for example, maternal) in somatic cells. In the sporadic form of retinoblastoma, he proposed that both mutations occur in somatic cells. It was further proposed that other tumor predisposition syndromes may follow similar rules. Knudson used a rat strain described by Eker that is predisposed to forming renal tumors (Eker and Mossige, 1961). Homozygous Eker mutants died embryonically, but when heterozygotes were subjected to ionizing radiation, they had a dose-dependent increase in renal tumors (Hino et al., 1993). The Eker rat mutation was eventually mapped to a *Tsc2* homolog (Hino et al., 1994; Yeung et al., 1994; Kobayashi et al., 1995). And *Tsc2* loss of heterozygosity (LOH), the genetic manifestation of the two-hit hypothesis, was detected in Eker renal tumors induced by mutagens and in *Tsc2* heterozygous mice (Kubo et al., 1994; Kobayashi et al., 1997; Hino et al., 2002; Ma et al., 2005). Subsequent studies confirmed that LOH, also referred to as biallelic inactivation, occurs in TSC patient tumors and malformations including SEGAs and cortical tubers (Chan et al., 2004; Crino et al., 2010; Qin et al., 2010a; Bongaarts et al., 2017; Martin et al., 2017).

**Figure 2:**
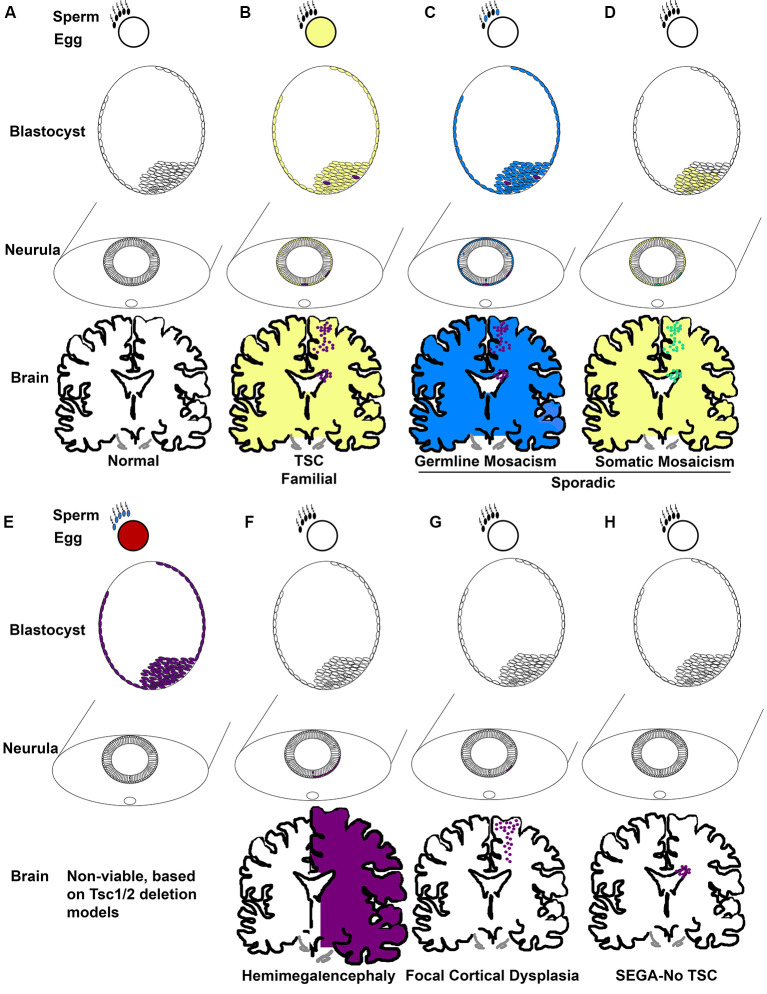
TSC genetic mechanisms of pathogenesis. **(A)** Development commences when gametes with wild-type TSC alleles fuse. Development proceeds to the blastocyst phase with pluripotent cells of the inner cell mass becoming sequentially restricted in fate. Epiblasts of the blastocyst form the ectoderm which eventually undergoes primary neurulation and forms the neural tube. Neuroepithelial cells (NECs) that line the neural tube are stem cells that generate NSCs. NSCs, for instance, radial glia, generate neurons, astrocytes, oligodendrocytes, and ependyma. **(B)** Familial TSC arises when a haploid germ cell carries a mutation in TSC1 or TSC2 (yellow). A second mutation resulting in loss of heterozygosity (LOH; purple) then is thought to arise sporadically at different times of development, for example in the blastocyst in cells that eventually generate the CNS. **(C)** Germline mosaicism is phenotypically similar to familial TSC with the exception that only the parent’s germ cells might carry TSC gene mutations due to a sporadic mutation during gametogenesis (blue) followed by a second LOH mutation (purple). **(D)** Somatic mosaicism arises when a sporadic mutation occurs in one allele (yellow) during development, for example in the blastocyst, and LOH (green) occurs in subsets of cells later. **(E)** Animal models demonstrate that germ cells without a functional copy of a TSC gene (red, blue) produce embryos with no functional TSC gene (purple) that are non-viable at mid-gestation. **(F)** Hemimegalencephaly occurs when mutations (orange) in TSC genes arise and affect one side of the developing brain. **(G)** Focal cortical dysplasia occurs when TSC gene mutations (orange) arise in radial glia that generates cells within the cortical plate. **(H)** SEGA in the absence of TSC is predicted to occur when TSC gene mutations (orange) arise in a cell of the SVZ, potentially, NSCs.

Taken together, TSC occurs in two forms, familial and sporadic. Familial occurs when one mutation is inherited from a parent and the second normal allele becomes mutated (Figures 2A,B). Of the 1/3 of patients with an inherited mutation, cases are more commonly caused by *TSC1* mutation (Dabora et al., 2001). Most TSC cases are not familial, they are sporadic (2/3; Dabora et al., 2001). Two mechanisms account for sporadic TSC. One mechanism is that patients (~3%) inherit mutations caused by gonadal/germ cell mosaicism in parents even though the parents do not carry mutations within the rest of their cells (Figure 2C; Verhoef et al., 1999). *De novo* germline mutations in *TSC1/2* are unknown and cannot be easily identified, thus the rates are likely higher than the 3% cited above. Sporadic TSC can also arise when a mutation occurs early in development in a stem cell that can give rise to many tissues. This is exemplified by the extreme case of TSC patients with somatic mosaicism (Tyburczy et al., 2015; Giannikou et al., 2019). The mutation of the second allele then is proposed to occur later in development within discrete cells (Figure 2D). The frequency that sporadic TSC is caused by germline vs. somatic mutations is not yet known regardless of evidence that both occur. Incidentally, familial *TSC2* mutations are less severe than sporadic *TSC2* mutations. One possible reason for the subtle phenotype of familial *TSC2* mutations is that severe mutations may not be compatible with life (Figure 2E).

The absence of an identified mutation in 10–15% of patients with a clinical diagnosis spurred studies that examined *TSC1/2* methylation and genetic modifiers and even the possibility of a *TSC3* gene (Niida et al., 2001; Dibble et al., 2012). However, an unidentified mutation is most likely caused by two effects. The first is that genetic tests focus on the 23 exons of *TSC1* and 41 exons of *TSC2*. Intronic mutations were identified in ~40% of patients which failed first level exonic Sanger sequencing and copy number variant/large deletion analysis (Tyburczy et al., 2015). Another cohort of these patients (58%) demonstrated genetic mosaicism (Tyburczy et al., 2015). Mosaicism is the phenomenon that differences in the genetic composition of cells that occur within a person and is caused by *de novo* mutations that arise in somatic cells (Nesbitt and Gartler, 1971). Thus, the timing of somatic cell mutation, the number of clones with *de novo* mutations, and the cells in which the mutation occurs contribute to the severity of TSC manifestations.

An example of this theoretical timing is that mutations in *TSC2* that cause hemimegalencephaly may occur in early neuroepithelial stem cells, those that occur later in embryonic radial glia may cause cortical tubers or focal cortical dysplasias, and later mutations in SVZ NSCs may cause SEGAs (Figures 2F–H; Henske et al., 1997; Chan et al., 2004; Crino et al., 2010; Qin et al., 2010a; D’Gama et al., 2017; Lim et al., 2017; Martin et al., 2017). Evidence of this phenomenon has historically been easier to detect in cells with a clonal origin that are in a confined location as occurs with tumors. Hence, somatic mutations in SEGAs were identified as occurring by LOH. Although the loss of heterozygosity was infrequently detected in cortical tubers, this may be caused by the cellular resolution of DNA sequencing. Previous experiments had taken specimens and isolated DNA from thousands of cells. Thus, in the background of a mosaic pattern of abnormal cells (think balloon cells, dysmorphic neurons, and giant cells) intermingled with wild-type cells, detection of LOH might be difficult. On the other hand, single-cell sequencing of cortical tubers has detected second hit mutations rendering cells with biallelic inactivation of either *TSC1* or *TSC2* (Crino et al., 2010). Thus, loss of both functional copies of *TSC1* or *TSC2* is likely sufficient but perhaps not necessary to cause TSC. Haploinsufficiency or dominant-negative mutations could also underlie pathogenesis. It is important to note that this is a limitation of deletion studies in the laboratory. Simple genetic deletion of a gene might not mimic the dominant-negative behavior of mutations in a clinical population and this is a current limitation of animal models and patient-derived induced pluripotent stem cells that use deletion to remove TSC genes.

Nevertheless, somatic mutations resulting in dominant-negative function would only require a single allele to be mutated to cause phenotypic changes. But because *TSC1* or *TSC2* and mTORC1 are required for embryogenesis, it seems likely that somatic biallelic inactivation may occur numerous times in different tissues. It would be interesting for example, to examine lesions from different regions in the same patient to determine whether the same mutations exist. It would also be interesting to determine in brain lesions whether when only a single allele is mutated whether this always corresponds to dominant-negative functions whereas two hits correspond to the loss of function mutations. And the evidence supports that mutations would likely differentially affect mTOR pathway activity meaning that some mutations would have more subtle and other more severe effects (Hoogeveen-Westerveld et al., 2011, 2012, 2013). Finally, studies have reported haploinsufficiency in mice alters network activity. These changes cause seizures by P16, learning deficits, and behavioral issues perhaps (Goorden et al., 2007; Ehninger et al., 2008; Auerbach et al., 2011; Lozovaya et al., 2014). Similarly, Eker rats have social behavioral defects, changes to episodic memory, and a predisposition to hyper-excitability (Rennebeck et al., 1998; Waltereit et al., 2006, 2011; Schneider et al., 2017). Subtle changes in mTORC1 activity may lead to abnormal axon targeting, inhibition of macroautophagy that prevents spine pruning, altered metabotropic glutamate receptor 5 dependent long term depression (Nie et al., 2010; Auerbach et al., 2011; Bartley et al., 2014; Tang et al., 2014). A limitation to these studies is that TSC heterozygous models may be subject to LOH during development leading to some of these changes.

### Treating TSC

Rapamycin is a macrolide compound generated by *Streptomyces hygroscopicus*, a resident of soil bacteria of the island of Rapa Nui, and was discovered for potent antifungal properties (Vézina et al., 1975). Rapamycin functions as an allosteric inhibitor through interactions with the protein FK506-binding protein (FKBP12) that promotes binding to and inhibition of mTORC1 signaling to p70 ribosomal S6 kinases (Chung et al., 1992; Brown et al., 1994; Sabatini et al., 1994; Sabers et al., 1995). The inhibition of mTORC1 by rapamycin is substrate selective (Kang et al., 2013). An example is that while rapamycin inhibits p70S6K-S6 signaling, inhibition of 4EBP phosphorylation is incomplete (Choo et al., 2008; Thoreen et al., 2009; Kang et al., 2013). Extended rapamycin treatment can also prevent the assembly of mTORC2 leading to reduced mTORC2 activity *in vitro* and *in vivo* (Sarbassov et al., 2006; Lamming et al., 2012). The assignment of mTOR functions based that rely only on rapamycin is therefore limited by the caveats that rapamycin inhibits mTORC2, partially inhibits selective substrate phosphorylation, and a third drawback that it activates a feedback loop by in which mTORC2 can become hyper-activated by rapamycin (Wan et al., 2007; Efeyan and Sabatini, 2010). A newer class of ATP competitive inhibitors exemplified by Torin1 demonstrates a more complete inhibition of mTORC1 substrate phosphorylation but also lacks specificity in that it also inhibits mTORC2 (Thoreen et al., 2009). From a mechanistic standpoint, using Torin1 has a limitation for selectively studying individual mTORCs, but from a clinical standpoint, Torin1-like compounds may be of greater utility in that it blocks both mTORCs and therefore has no feedback to mTORC2. Nevertheless, many of the key findings on mTOR based on rapamycin treatment have been affirmed with Torin1, such as the role of mTOR regulation of 5’-TOP translation (Thoreen et al., 2012).

Numerous studies have demonstrated the utility of rapamycin in reducing the cellular and neuropathophysiological manifestations in animal models of TSC. Notable examples include administration of rapamycin in postnatal day seven in *synapsin*-CRE mice having *Tsc1* deleted from neurons reduces p70S6 kinase activity, neuron size, dendritic spine density, cortical thickness, and increases survival (Meikle et al., 2008). A single dose of rapamycin administered to pregnant *nestin*-CRE *Tsc1* mice or supplemented with a postnatal day three injection also enhanced survival (Anderl et al., 2011). Continual treatment from postnatal days 8–60 exerted similar effects on the survival of *Emx-*CRE *Tsc1* deleted mice and even ameliorated seizures (Magri et al., 2011). Surprisingly, while numerous animal studies show that rapamycin reduces TSC cellular and neuropathophysiological manifestations, genetic studies have not yet clarified the potential mechanism(s) (mTORC1 knockout vs. mTORC2 knockout). This is particularly fascinating because TSC is also characterized by altered mTORC2 pathway activity and mTORC2 is implicated in mediating behavioral and neurophysiological changes in mice having neuron PTEN deletion, which mimics the TSC-related disorders Cowden Syndrome and Lhermitte-Duclos disease (Chen et al., 2019).

Evidence for the utility of rapamycin in TSC was first demonstrated when the SEGAs of four TSC patients regressed following rapamycin treatment (Franz et al., 2006). Clinical trials demonstrated that the rapamycin analog (rapalog) everolimus was successful in shrinking TSC SEGAs by 30–50% within 6 months and reducing seizure burden (Krueger et al., 2013a). Long-term treatment for up to 3 years successfully reduced SEGAs by 56% and prevented the formation of new SEGAs (Franz et al., 2013). EXIST (EXamining everolimus In a Study of TSC) phase III clinical trials further indicated that everolimus markedly reduced seizure frequency (French et al., 2016). Major limitations to rapalogs are that the effects are reversible, may not effectively eliminate neurological manifestations, and have side effects including immunosuppression (Franz and Capal, 2017). Most relevant is that treatment with rapalogs is a life-long sentence because treatment cessation will likely be associated with the return of symptoms (Franz and Capal, 2017) Thus, active areas of study include determining whether ATP-pocket mTOR inhibitors permanently alleviate symptoms.

### Summary

Clinical observation, genetic analysis, and laboratory research have assisted in developing a robust understanding of the neuropathology of TSC. There remain questions as to the extent that hamartin and tuberin regulate mTORC1-independent pathways that contribute to TSC pathology (Zhang et al., 2014, 2020). However, the importance of the mTOR pathway is underscored by the clinical utility of rapamycin-like compounds that inhibit mTORC1 (Franz and Capal, 2017). Incidentally, the role of signals that regulate hamartin and tuberin activity as well as the mTORC1 substrates responsible for specific developmental events are only now being determined. Perhaps most striking is the fact that somatic mutations that cause mTORC1 pathway activation have been identified as a cause of a plethora of neurological diseases (Lee et al., 2012; Poduri et al., 2012; Parker et al., 2013; Lal et al., 2014; Baek et al., 2015; Baulac et al., 2015; Crino, 2015; D’Gama et al., 2015, 2017; Leventer et al., 2015; Lim et al., 2015; Baulac, 2016; Korenke et al., 2016; Møller et al., 2016; Hanai et al., 2017; Park et al., 2018; Iffland and Crino, 2019; Kim et al., 2019; Pelorosso et al., 2019; Salinas et al., 2019; Zhao et al., 2019; Garcia et al., 2020). Thus, what has been learned by studying the TSC pathway may now be applied to an expanding number of patients.

## Author Contributions

DF conceived, wrote, and edited the article in its entirety.

## Conflict of Interest

The author declares that the research was conducted in the absence of any commercial or financial relationships that could be construed as a potential conflict of interest.
